# Exercise-mediated renoprotection across kidney diseases: emerging roles of microRNAs

**DOI:** 10.3389/fphys.2026.1981071

**Published:** 2026-10-28

**Authors:** Weihao Hong, Bowen Dong, Haibin Yu, Binbin Fang

**Affiliations:** School of Physical Education, Quanzhou Normal University, Quanzhou, China

**Keywords:** exercise, exercise-miRNA-kidney axis, kidney diseases, microRNA, renal protection

## Abstract

Exercise exerts renoprotective effects across diverse kidney disease settings, yet the molecular mechanisms underlying these adaptations remain incompletely understood. MicroRNAs (miRNAs), owing to their capacity to regulate multiple targets within interconnected signaling networks, may serve as a regulatory interface between exercise stimuli and renal adaptation. This narrative review synthesizes current understanding of the pathophysiological roles of miRNAs in kidney diseases and emerging evidence linking exercise-associated miRNA remodeling to renoprotection. Particular attention is given to miRNAs involved in metabolic and organelle homeostasis, inflammation and oxidative stress, regulated cell death, and fibrotic remodeling, as well as to exercise-responsive miRNAs detected in renal tissue, extracellular vesicles (EVs), and the circulation. Across experimental models, several candidate pathways, including miR-34a–Klotho, miR-296-5p–SGLT2, and miR-126/Nrf2, show coordinated changes in miRNA expression, related signaling pathways, and renal outcomes. Beyond renal tissue, EV-associated miRNAs suggest a potential route for muscle-kidney communication, while circulating miRNAs may provide biomarkers of systemic responses to exercise. Overall, miRNAs represent an emerging component of the molecular network potentially linking exercise to improved renal outcomes across kidney disease settings, although their specific mechanistic contributions remain to be established.

## 1 Introduction

Kidney diseases arise from diverse metabolic, hemodynamic, ischemic, toxic, immune-mediated, and aging-related insults. Despite their heterogeneous etiologies, many forms of kidney injury converge on a common pathological network characterized by disrupted cellular metabolism, persistent stress signaling, progressive cell loss, and maladaptive tissue remodeling. The interaction of these processes gradually destabilizes glomerular and tubular homeostasis, ultimately impairing renal structure and function (Webster et al., 2017; Gewin, 2018; Tang et al., 2021).

Exercise has emerged as a promising non-pharmacological strategy for renal protection. Across several experimental kidney disease settings, regular exercise alleviates systemic metabolic and hemodynamic stress while promoting adaptive responses within the kidney. These effects are accompanied by improved mitochondrial function, reduced tissue injury, and attenuation of progressive remodeling (Heiwe and Jacobson, 2014; Johansen and Painter, 2012; Yamakoshi et al., 2021; Kosaki et al., 2019; Monno et al., 2021; Hong et al., 2024). Clinical studies support the feasibility of exercise and gains in physical capacity, although effects on albuminuria and kidney function remain variable (Leehey et al., 2009, 2016). Because such benefits arise from coordinated changes across multiple biological systems, they are unlikely to depend on a single downstream pathway. The regulatory mechanisms that integrate exercise stimuli into a coherent renal response, however, remain incompletely understood.

MicroRNAs (miRNAs) are plausible components of this regulatory framework because individual miRNAs can simultaneously modulate multiple genes within interconnected signaling networks (Bartel, 2018; O'Brien et al., 2018). Their established involvement in kidney injury and repair provides a biological basis for examining whether miRNAs contribute to exercise-mediated renoprotection (Mahtal et al., 2022). Studies of exercise-associated miRNA remodeling have identified changes across renal tissue and extra-renal compartments, including the circulation and extracellular vesicles (EVs), suggesting that miRNAs may link local renal adaptation with systemic and interorgan responses to exercise (Sapp et al., 2017; Baggish et al., 2011; Uhlemann et al., 2014; Barber et al., 2019; Whitham et al., 2018; Frühbeis et al., 2015; Safdar et al., 2016; Sisia et al., 2026). However, the available evidence remains largely associative. Kidney-focused studies generally report altered miRNA abundance alongside improved renal phenotypes, whereas studies of circulating and EV-associated miRNAs primarily characterize systemic exercise responses, with limited evidence of renal delivery or function.

To contextualize these emerging findings, this narrative review outlines the major pathophysiological roles of miRNAs in kidney disease and considers how exercise-associated miRNA remodeling may intersect with renal metabolic, inflammatory, cytoprotective, and fibrotic responses, including potential systemic and interorgan contributions. Studies directly linking exercise, miRNAs, and renal outcomes are additionally classified according to the directness of mechanistic evidence. This integrated perspective may help refine the current conceptual framework of the exercise–miRNA–kidney axis and highlight priorities for future mechanistic and translational investigation.

## 2 Review methodology and evidence classification

Literature searches were conducted in PubMed, Web of Science, and Scopus from database inception through October 6, 2026 to identify studies relevant to exercise, miRNAs, and kidney health or disease. Search strategies combined terms related to exercise (“exercise”, “physical activity”, “aerobic exercise”, “aerobic training”, “resistance exercise”, “resistance training”), miRNAs (“microRNA”, “miRNA”), and renal conditions or outcomes (“kidney”, “renal”, “chronic kidney disease”, “CKD”, “diabetic kidney disease”, “DKD”, “acute kidney injury”, “AKI”, “renal fibrosis”, and “renal aging”). Additional searches incorporating “extracellular vesicle” or “exosome” were performed to capture studies investigating EV-associated miRNAs. Reference lists of relevant original articles and reviews were also screened for potentially eligible studies. No language restrictions were imposed.

A focused structured search was used to identify primary studies informing the proposed exercise–miRNA–kidney axis. To be included in this core evidence set, studies were required to examine, within the same study, (1) an exercise intervention or exercise exposure, (2) one or more miRNAs, and (3) a renal-related outcome. Renal outcomes included measures of kidney function, urinary injury markers, renal histopathology, fibrosis, inflammation, oxidative stress, or other molecular or cellular indices related to renal injury. Studies reporting exercise-associated miRNA changes without a renal outcome, as well as studies linking individual miRNAs to renal disease in the absence of an exercise component, were not considered direct evidence. Studies meeting these criteria were summarized in **Table 1** and classified using a three-level framework according to the directness of exercise-related mechanistic evidence. Level A required miRNA-specific manipulation to alter the renal response to exercise, supported by target validation and rescue or equivalent mechanistic confirmation. Level B comprised same-study observations linking exercise-associated miRNA changes, a related target or pathway, and renal outcomes without demonstrating miRNA-specific mediation. Level C comprised concurrent assessment of miRNAs and renal outcomes without exercise-linked miRNA target-pathway validation. The classification reflects the directness of evidence for the proposed exercise-miRNA-kidney axis. It does not assess the overall methodological quality or risk of bias of individual studies.

**Table 1:** Evidence classification for the proposed exercise–miRNA–kidney axis.

Evidence level	Studies assigned	Basis for classification
A: Direct causal mediation	None identified	No study combined an exercise intervention, miRNA-specific manipulation, renal outcomes, and target-rescue or equivalent mechanistic validation.
B: Same-study pathway support	Geng et al., 2022; Asgari et al., 2022; Zhao et al., 2024; Zhao et al., 2023; Mirtajaddini Goki et al., 2026	Exercise altered a miRNA together with a related target or downstream pathway and renal outcomes in the same study.
C: Concurrent association	Luan et al., 2025; Jiang et al., 2025	Exercise-associated miRNA changes were assessed together with renal outcomes, without demonstrating exercise-linked miRNA target-pathway validation.

## 3 Pathophysiological roles of miRNAs in kidney diseases

Kidney diseases of diverse etiologies converge on interconnected pathological processes, including metabolic and organelle dysfunction, inflammation, oxidative injury, regulated cell death, and maladaptive repair. The sustained interaction of these processes disrupts glomerular and tubular homeostasis and promotes the transition from reversible injury to chronic structural and functional deterioration. Through the coordinated post-transcriptional regulation of multiple target genes, miRNAs influence renal stress responses across different stages of disease progression (Mahtal et al., 2022) (**Table 2**).

**Table 2:** Representative pathophysiological roles of miRNAs across kidney disease models.

Study	Experimental or clinical model	miRNA/target or pathway	Principal finding
Chau et al., 2012	UUO and unilateral renal IRI mice; primary renal epithelial cells	miR-21–PPARα/Mpv17l	miR-21 suppressed metabolic pathways, impaired fatty acid oxidation, and promoted oxidative injury and renal fibrosis.
Morrison et al., 2017	HFD-fed human-CRP transgenic mice	miR-21	Renal miR-21 expression was associated with KIM-1 expression and fibrotic kidney injury.
Liu et al., 2015	Experimental renal IRI	miR-21–Rab11a	miR-21 disrupted endosomal trafficking and autophagic flux, thereby aggravating renal IRI.
Chen, 2022	Experimental diabetic kidney disease and high-glucose-exposed renal tubular cells	circ_000166–miR-296–SGLT2	miR-296 overexpression or SGLT2 silencing attenuated fibrotic and apoptotic responses.
Abdollahi et al., 2022	HFD-fed mice with genetic miR-379 deletion	miR-379–EDEM3	miR-379 deletion attenuated glomerular injury, albuminuria, inflammation, and fibrosis.
Kato et al., 2021	Experimental DKD in wild-type and miR-379-deficient mice	miR-379–FIS1	miR-379 deletion enhanced adaptive mitophagy and ameliorated diabetic kidney injury.
Eritja et al., 2024	HFD-fed mice	Multiple renal miRNAs	Renal miRNA profiling implicated PI3K–AKT, mTOR, adipocytokine, and lipid-metabolism pathways.
López-Martínez et al., 2024	HFD-fed Wistar rats	miR-205 and related miRNAs–PTEN	HFD feeding induced albuminuria, mesangial expansion, podocyte hypertrophy, and podocyte lipid accumulation alongside renal and urinary miRNA changes.
Zheng et al., 2019	HFD-fed C57BL/6J mice	miR-155–SHIP1/NF-κB	miR-155 promoted renal inflammation, macrophage recruitment, albuminuria, and functional impairment.
Sun et al., 2019	HFD-fed mice and serum from subjects with obesity	miR-802–NF-κB-repressing factor	miR-802 enhanced inflammatory signaling and renal injury by suppressing NF-κB-repressing factor.
Amrouche et al., 2017	Human renal samples and experimental ischemic AKI	miR-146a–CXCL8/IRAK1/TRAF6	miR-146a constrained inflammatory signaling and limited ischemic kidney injury.
Dai et al., 2016	LPS-preconditioned mice with renal IRI	miR-146a–IRAK1/TRAF6	miR-146a-mediated negative feedback was required for LPS-induced protection against renal IRI.
Sun et al., 2022	Experimental renal IRI	EV-miR-223-3p–NLRP3	EV-delivered miR-223-3p promoted mitophagy and suppressed NLRP3 inflammasome activation and tubular injury.
Liu et al., 2021	Urografin-treated HK-2 cells	miR-30e-5p–Beclin 1	miR-30e-5p suppressed autophagy and promoted tubular-cell apoptosis by targeting Beclin 1.
Ding et al., 2020	Experimental renal IRI	miR-182-5p–GPX4; miR-378a-3p–SLC7A11	Both miRNAs increased ferroptotic susceptibility by suppressing key components of the antioxidant defense system.
Liu et al., 2019	Experimental renal fibrosis	miR-34a–Klotho	miR-34a suppressed Klotho and promoted fibrotic remodeling.
Liang et al., 2021	Experimental DKD	p53–miR-34a–SIRT1	Inhibition of the p53–miR-34a–SIRT1 pathway attenuated podocyte injury.
Bai et al., 2011	Aged rats and senescent renal cells	miR-34a/miR-335–mitochondrial antioxidant enzymes	miR-34a and miR-335 impaired mitochondrial antioxidant defenses and promoted renal senescence.
Qin et al., 2011	Experimental renal fibrosis	TGF-β/Smad3–miR-29	TGF-β/Smad3-mediated suppression of miR-29 increased extracellular matrix production and renal fibrosis.
Kato et al., 2007	Diabetic mouse glomeruli and cultured mesangial cells	TGF-β–miR-192–ZEB1/ZEB2	TGF-β-induced miR-192 suppressed ZEB1 and ZEB2 and enhanced collagen expression.
Putta et al., 2012	Experimental DKD	miR-192/TGF-β signaling	Pharmacological inhibition of miR-192 reduced TGF-β signaling, extracellular matrix accumulation, and renal fibrosis.
Krupa et al., 2010	Human diabetic-nephropathy biopsies and cultured proximal tubular cells	Reduced miR-192	Reduced miR-192 expression was associated with increased fibrogenesis, demonstrating its context-dependent role.
Li et al., 2020	UUO rats and high-glucose-exposed HK-2 cells	miR-542-3p–AGO1	miR-542-3p suppressed AGO1 and promoted tubular-cell activation and tubulointerstitial fibrosis.
Zhou et al., 2021	Unilateral renal IRI in mice and hypoxic tubular cells	Tubular EV-miR-150-5p–SOCS1	Tubular-cell-derived EV miR-150-5p suppressed SOCS1, activated interstitial fibroblasts, and promoted renal fibrosis.

### 3.1 miRNAs regulating metabolic and organelle homeostasis

Renal cells depend on coordinated substrate utilization, mitochondrial ATP production, endoplasmic reticulum proteostasis, and autophagic clearance. In metabolic and diabetic kidney diseases, excess glucose and lipid availability promote renal lipid uptake and lipogenesis while impairing mitochondrial fatty acid oxidation. The resulting lipotoxicity disrupts mitochondrial function, increases reactive oxygen species production, induces endoplasmic reticulum stress, and compromises lysosomal and autophagic homeostasis (Webster et al., 2017).

miR-21 links metabolic reprogramming with organelle dysfunction. In hypoxia-exposed renal epithelial cells and unilateral ureteral obstruction (UUO), miR-21 suppressed peroxisome proliferator-activated receptor-α (PPARα)-dependent metabolic networks and the mitochondrial protein Mpv17l, thereby impairing fatty acid oxidation and promoting lipid accumulation and oxidative injury (Chau et al., 2012). Increased renal miR-21 has also been associated with kidney injury molecule-1 (KIM-1) expression and fibrosis in high-fat diet (HFD)-fed mice (Morrison et al., 2017). In ischemia–reperfusion injury (IRI), miR-21 additionally repressed Rab11a, disrupted recycling-endosome trafficking, and impaired autophagic flux, whereas miR-21 inhibition restored Rab11a expression and autophagic activity (Liu et al., 2015). These findings implicate miR-21 in the coordinated disruption of energy metabolism and intracellular quality control.

Renal glucose transport represents another metabolic process subject to miRNA regulation. In experimental diabetic kidney disease, miR-296 was implicated in sodium-glucose cotransporter 2 (SGLT2) regulation, with circ_000166-mediated suppression of miR-296 associated with increased SGLT2 expression. Conversely, miR-296 overexpression or SGLT2 silencing attenuated fibrotic and apoptotic responses (Chen, 2022).

miR-379 connects metabolic stress with endoplasmic reticulum dysfunction. In HFD-fed mice, increased glomerular miR-379 suppressed endoplasmic reticulum degradation-enhancing α-mannosidase-like protein 3 (EDEM3), whereas miR-379 deletion restored EDEM3 expression and attenuated albuminuria, glomerular hypertrophy, structural injury, profibrotic gene expression, and interstitial fibrosis (Abdollahi et al., 2022). In DKD, miR-379 deletion has also been reported to enhance mitochondrial fission 1 protein (FIS1)-dependent adaptive mitophagy (Kato et al., 2021). High insulin similarly induced miR-379 and reduced EDEM3 in mesangial cells, supporting a hyperinsulinemia–miR-379–EDEM3 pathway in metabolic kidney injury.

Profiling studies further suggest that metabolic kidney injury involves broader miRNA networks. In HFD-fed mice, changes in miR-5099, miR-551b-3p, and miR-146a-3p were linked by target prediction to phosphoinositide 3-kinase–protein kinase B (PI3K–AKT), adipocytokine, mechanistic target of rapamycin (mTOR), and fatty acid metabolic pathways (Eritja et al., 2024). In HFD-fed rats, increased renal and urinary miR-205, together with miR-22-3p, miR-22-5p, and miR-144-3p, was associated with reduced glomerular phosphatase and tensin homolog (PTEN) and enhanced podocyte lipid uptake and vacuolization (López-Martínez et al., 2024).

### 3.2 miRNAs modulating inflammatory and redox signaling

Metabolic, ischemic, and toxic insults activate Toll-like receptor, nuclear factor-κB (NF-κB), and inflammasome pathways in renal endothelial, epithelial, and immune cells, promoting cytokine production and leukocyte recruitment. miRNAs regulate both activating and inhibitory components of these pathways and may therefore amplify inflammatory injury or reinforce endogenous negative-feedback mechanisms.

miR-155 is a functional promoter of obesity-related renal inflammation. HFD feeding and palmitic acid exposure increased miR-155 in renal tissue and vascular endothelial cells, respectively. miR-155 directly suppressed SH2 domain-containing inositol 5-phosphatase 1 (SHIP1), thereby enhancing NF-κB activation, cytokine production, endothelial dysfunction, macrophage recruitment, and renal lipid accumulation. Kidney-directed delivery of a miR-155 sponge restored SHIP1 and attenuated albuminuria, inflammatory infiltration, and structural injury, whereas SHIP1 silencing abolished these effects (Zheng et al., 2019). These findings support a functional miR-155–SHIP1–NF-κB axis in metabolic kidney injury.

miR-802 promotes renal inflammation through suppression of another endogenous inhibitor of NF-κB signaling. In HFD-fed mice, increased renal miR-802 directly repressed NF-κB-repressing factor, facilitating inhibitor of κB (IκB) phosphorylation, cytokine production, and macrophage recruitment. Kidney-directed miR-802 silencing restored NF-κB-repressing factor and reduced glomerular enlargement, basement-membrane thickening, collagen deposition, and renal dysfunction (Sun et al., 2019). Increased renal miR-802-5p in an independent HFD study further supports its association with obesity-related kidney injury (Luan et al., 2025).

Conversely, miR-146a and miR-223-3p may constrain inflammatory signaling. miR-146a represses interleukin-1 receptor-associated kinase 1 (IRAK1) and tumor necrosis factor receptor-associated factor 6 (TRAF6) as part of a Toll-like receptor/NF-κB negative-feedback loop and has been associated with reduced inflammation and improved recovery after ischemic AKI (Amrouche et al., 2017; Dai et al., 2016). miR-223-3p similarly limits thioredoxin-interacting protein (TXNIP)/NOD-like receptor family pyrin domain-containing 3 (NLRP3)-dependent inflammasome activation in tubular and ischemia–reperfusion models (Sun et al., 2022). Renal inflammation may therefore reflect the balance between pathogenic miRNA activity and endogenous counter-regulatory miRNA responses.

### 3.3 miRNAs governing regulated cell death

Persistent metabolic, organelle, inflammatory, and oxidative stress can overwhelm renal adaptive capacity and promote regulated cell death. miRNAs influence this transition by modulating autophagy, apoptosis, ferroptosis, antioxidant defense, and cellular senescence.

miR-30e-5p links defective autophagy with tubular apoptosis. In contrast-exposed tubular cells, increased miR-30e-5p suppressed Beclin1, reduced autophagic activity, and increased caspase-3 expression and DNA fragmentation (Liu et al., 2021). miR-30e-5p inhibition restored Beclin1-dependent autophagy and reduced apoptosis, indicating that this miRNA compromises an upstream survival mechanism rather than acting exclusively on terminal apoptotic pathways.

miR-182-5p and miR-378a-3p regulate ferroptotic susceptibility by targeting glutathione peroxidase 4 (GPX4) and solute carrier family 7 member 11 (SLC7A11), respectively. In renal IRI, suppression of these antioxidant systems increased iron accumulation, lipid reactive oxygen species, mitochondrial superoxide, and tubular cell death. Inhibition of either miRNA restored its corresponding target and attenuated ferroptotic injury (Ding et al., 2020). Renal miR-182-5p is also increased following HFD exposure, making it a biologically plausible candidate in metabolic kidney injury, although GPX4-dependent regulation has not been established in that context (Luan et al., 2025).

miR-34a may contribute to aging-related renal injury through pathways linking cellular senescence, mitochondrial dysfunction, and fibrosis. Activation of the p53/miR-34a axis has been associated with reduced Klotho- and sirtuin 1 (SIRT1)-related protective signaling, increased susceptibility to apoptosis, and impaired mitochondrial homeostasis (Liu et al., 2019; Liang et al., 2021; Bai et al., 2011). In aged kidneys, these changes coincide with activation of transforming growth factor-β (TGF-β)/Smad and Wnt/β-catenin signaling, suggesting that miR-34a may connect senescence-associated stress with subsequent maladaptive remodeling.

### 3.4 miRNAs shaping maladaptive repair and fibrotic remodeling

Persistent epithelial injury, inflammation, capillary dysfunction, and cellular senescence promote fibroblast activation and excessive extracellular matrix deposition. miRNAs regulate this transition through effects on TGF-β signaling, matrix-gene expression, fibroblast activation, and tubular–stromal communication.

The miR-29 family functions as an antifibrotic regulator by directly repressing multiple extracellular matrix transcripts. TGF-β1/Smad3 signaling suppresses miR-29, thereby relieving post-transcriptional inhibition of collagen and other matrix-associated genes. Conversely, restoration of miR-29 limits matrix synthesis and renal fibrosis (Qin et al., 2011).

miR-192 also participates in TGF-β-dependent matrix regulation, although its effects are cell- and context-dependent. In diabetic mesangial cells, TGF-β-induced miR-192 represses zinc finger E-box-binding homeobox 1 and 2 (ZEB1 and ZEB2), promoting collagen expression and downstream miR-200 signaling (Kato et al., 2007). miR-192 inhibition has reduced TGF-β, collagen, and fibronectin expression in experimental DKD (Putta et al., 2012). However, opposite expression patterns in other renal settings indicate that its biological effects vary according to cell type, disease stage, and injury context (Krupa et al., 2010).

miR-542-3p promotes tubulointerstitial fibrosis through direct repression of Argonaute 1 (AGO1). Its upregulation in unilateral ureteral obstruction and high-glucose-exposed tubular cells increased α-smooth muscle actin (α-SMA), collagen, and fibronectin expression, whereas miR-542-3p inhibition restored AGO1 and attenuated fibrotic changes (Li et al., 2020).

Fibrotic signaling may also be propagated through extracellular vesicles. Following unilateral IRI, hypoxic tubular epithelial cells released miR-150-5p-enriched vesicles that transferred miR-150-5p to interstitial fibroblasts. Subsequent suppressor of cytokine signaling 1 (SOCS1) suppression increased α-SMA, fibronectin, and collagen production. Inhibition of vesicle release, depletion of vesicular miR-150-5p, or restoration of SOCS1 reduced fibroblast activation and renal fibrosis (Zhou et al., 2021). This pathway demonstrates how miRNAs can mediate communication between injured tubular cells and neighboring stromal cells.

## 4 Emerging roles of miRNAs in exercise-mediated renoprotection

Exercise-mediated renoprotection involves coordinated improvements in metabolic regulation, mitochondrial function, redox balance, inflammation, cell survival, and fibrotic remodeling. Emerging evidence indicates that changes in miRNA expression accompany several of these adaptations and may contribute to the regulation of relevant molecular pathways (**Figure 1**, **Table 3**).

**Figure 1:**
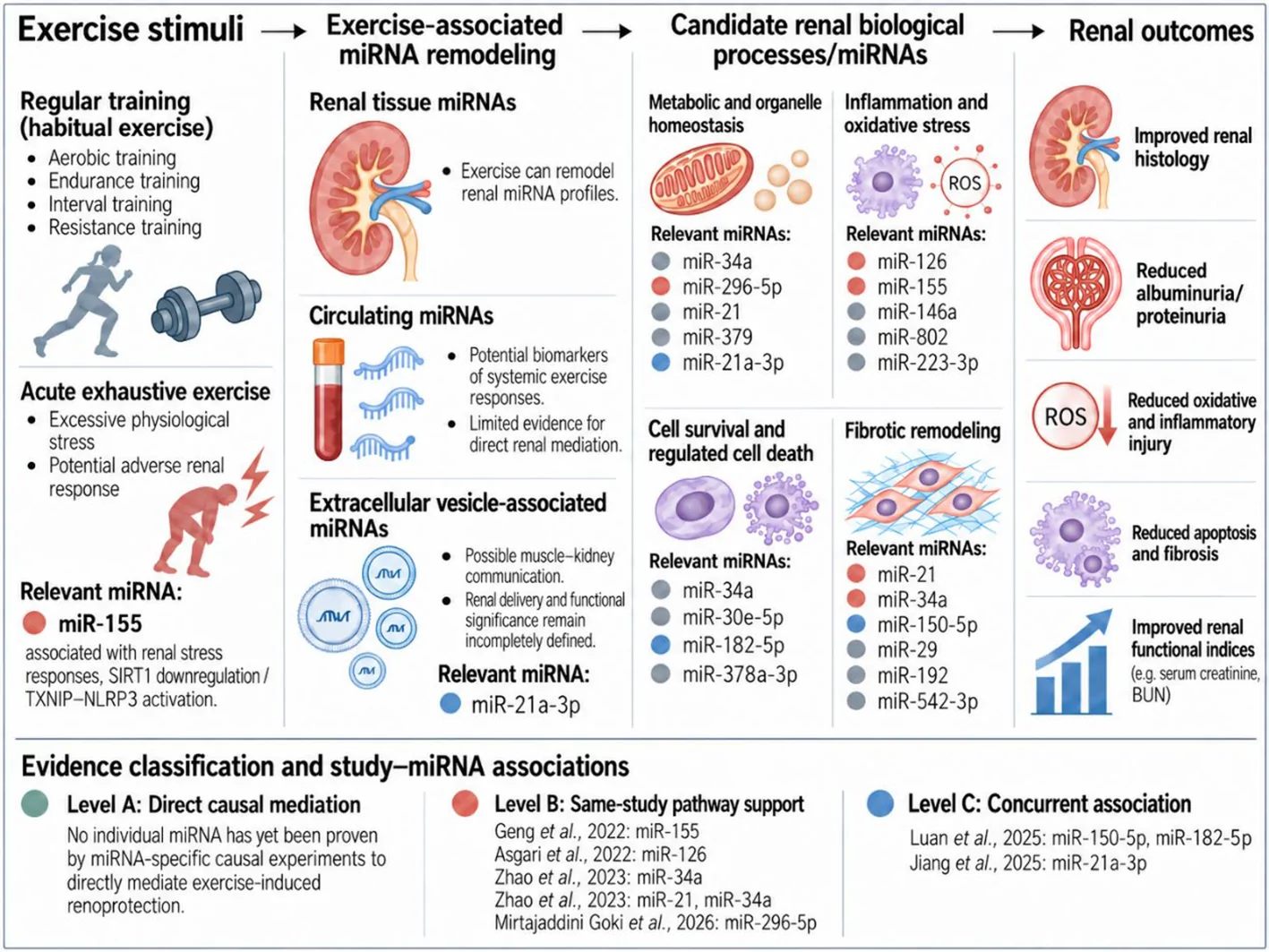
Conceptual framework linking exercise-associated miRNA remodeling to renoprotection. Regular exercise may promote renal adaptation, whereas acute exhaustive exercise can induce renal stress responses involving miR-155. Exercise-associated miRNA remodeling occurs in renal tissue, the circulation, and EVs, with potential relevance to metabolic and organelle homeostasis, inflammation and oxidative stress, cell survival and regulated cell death, and fibrotic remodeling. These processes may be associated with improvements in renal structure and function and attenuation of kidney injury. Colored markers indicate the current evidence levels: red, Level B (same-study pathway support); blue, Level C (concurrent association); and green, Level A (direct causal mediation), for which no qualifying studies have been identified. Gray markers denote miRNAs implicated in renal biological processes by non-exercise studies but whose roles in exercise-mediated renoprotection remain unverified. ROS, reactive oxygen species; BUN, blood urea nitrogen.

**Table 3:** Renal and systemic miRNAs in exercise-related contexts.

Study	Study population/model	Exercise protocol and sampling	miRNA/compartment	Main finding and renal relevance
I. Renal miRNA responses to exercise				
Geng et al., 2022	Healthy male Sprague–Dawley rats	Acute exhaustive treadmill exercise; immediate post-exercise sampling.	Serum and renal miR-155↑	Increased miR-155 accompanied SIRT1 reduction, TXNIP/NLRP3 activation, and acute renal injury.
Luan et al., 2025	HFD-induced obesity in male C57BL/6 mice	Treadmill training after 12-week HFD exposure: ~60% VO_2_max, 45 min/session, 5 days/week, 8 weeks; urine collected overnight and blood/kidneys the next day after the final session.	Renal miR-150-5p↓ and miR-182-5p↓	miRNA remodeling accompanied reduced proteinuria, oxidative injury, apoptosis, inflammation, and fibrosis.
Asgari et al., 2022	Diabetic rats with myocardial I/R; exercise, *Crataegus persica* extract or combined treatment	Interval training after diabetes induction and before myocardial I/R: 10 × 3 min at 75% maximal speed, with recovery at 50%, 5 days/week, 10 weeks; kidneys assessed after 24-h reperfusion.	Renal miR-126/Nrf2-related signaling	Exercise alone increased miR−126/Nrf2 expression and reduced histological injury. Combined treatment also reduced injury and improved selected redox indices; extract alone did not improve histology.
Zhao et al., 2024	Young and aged male C57BL/6J mice (2 and 19 month)	Treadmill training: 41 min/session, 5 days/week, 12 weeks (main workload: 14 m/min, 6% incline); blood/kidneys collected after a 12-h post-training fast.	Renal miR-34a↓	Reduced miR-34a accompanied Klotho restoration, suppression of profibrotic signaling, and reduced renal fibrosis.
Zhao et al., 2023	Naturally aged rats (21 months)	Moderate swimming training for 12 weeks.	Renal miR-21 and miR-34a	miRNA remodeling coincided with restored PPARα and reduced creatinine/BUN, histological injury, fibrosis, lipid accumulation, oxidative stress, and inflammation.
Mirtajaddini Goki et al., 2026	Male Wistar rats with D-galactose-induced aging and controls	MIET or HIIT during D-galactose-induced aging.	Renal miR-296-5p↑	Both training modalities increased miR−296−5p, reduced SGLT2 protein, and attenuated glomerulosclerosis. HIIT also normalized BUN and creatinine.
II. EV-Mediated interorgan communication				
Jiang et al., 2025	Male C57BL/6J mice; complementary human muscle and EV studies	Mice: single 1-h RotaRod bout at 5.8 m/min; sampling 15 min afterward. Humans: resistance training, 1 set at 10RM, 3 sessions/week, 12 weeks, plus Korean mistletoe extract.	Muscle/sEV miR-21a-3p	Damaged-muscle sEVs aggravated renal injury through miR−21a−3p-related effects. Post-exercise mouse EVs and human combined-intervention EVs reduced profibrotic gene expression in renal tubular cells
Zhang et al., 2018	Healthy and streptozotocin-diabetic male C57BL/6J mice	NA	Serum exosomal and renal miR-23a/27a↑	Intramuscular overexpression increased serum exosomal and renal miRNAs and reduced BUN and renal fibrosis. The findings support muscle–kidney communication.
Wang et al., 2019	Male C57BL/6J mice with UUO	NA	Exosomal and renal miR-29	RVG-modified, miR−29-containing exosomes delivered from muscle increased renal miR−29 and reduced fibrosis, with validation of YY1 and TGF-β3 targeting
Ji et al., 2023	Male *db/db* mice (12–20 weeks during treatment)	NA	EV miR-23a/27a/26a cluster	Exosomes accumulated in injured kidneys and reduced tubular injury and tubulointerstitial fibrosis. Target validation implicated *Lpp*, *Zbtb20*, and *Klhl42*
III. Circulating and urinary miRNAs in exercise responses				
Van Craenenbroeck et al., 2015	Acute cohort: nondialysis CKD patients and controls. Training cohort: stage 3–4 CKD patients	Acute cycling CPET: plasma collected before and 10 min afterward. Cycling training: 4 × 10 min/day, 12 weeks, at 90% of heart rate at anaerobic threshold; resting and CPET sampling repeated after training.	Plasma miR-150, miR-146a, miR-125b, and miR-210	Circulating miRNAs showed acute exercise responses and altered exercise responsiveness after training. Resting levels were not significantly changed by training
Van Craenenbroeck et al., 2016	Nondialysis CKD patients (stages 1–5) and controls; cross-sectional study	NA; CPET assessed exercise capacity rather than a training intervention.	Circulating miR-146a and miR-150	Both miRNAs correlated with eGFR, arterial stiffness, and peak oxygen uptake. Only the exercise-capacity association was lost after adjustment for vascular function
Spakova et al., 2020	Hemodialysis patients completing follow-up	Intradialytic resistance training: 40 min/session, 3 sessions/week, 12 weeks (Borg 11–14); plasma miRNAs measured at baseline only.	Circulating miR-206 and miR-23a	Baseline miR−206 predicted strength gains in trained patients; miR−23a was associated with inactivity-related functional changes.
de Luca Corrêa et al., 2022	Older hemodialysis patients (mean age 68.3 years)	Resistance training: 3 sessions/week, 24 weeks; blood collected before and after the intervention.	Circulating miR-1↑ and miR-31↓	miRNA changes accompanied improvements in physical performance, inflammation, sestrin-2, and nitric oxide-related measures.
Park and Moon, 2022	Healthy men (mean age 25.5 years)	Acute exhaustive 20-m shuttle run; urine collected before exercise (first morning sample), immediately afterward, and after 1 h of recovery.	Urinary EV-miRNAs	Urinary EV abundance increased immediately after exercise and returned to baseline after 1 h; miRNA cargo changed across sampling times.
Pietrangelo et al., 2023	Male triathletes; cross-sectional study	NA; cross-sectional comparison of habitual training status. First-morning urine collected following a day without training.	Urinary EV-miRNAs	Urinary EV characteristics and metabolic miRNA profiles differed according to habitual endurance-training status.

HIIT, high-intensity interval training; MIET, moderate-intensity endurance training; CPET, cardiopulmonary exercise testing; RM, repetition maximum; VO_2_max, maximal oxygen uptake; NA, not applicable; ↑, increased; ↓, decreased.

### 4.1 Renal miRNA responses to exercise in health

Studies in healthy animals provide a physiological reference for interpreting renal miRNA responses in kidney diseases. In healthy rats, six weeks of sustained swimming altered 17 renal miRNAs, comprising 10 upregulated and 7 downregulated miRNAs (Junling et al., 2024). Predicted targets were enriched in thyroid and Hippo signaling pathways, suggesting that miRNA remodeling may form part of normal renal adaptation to sustained exercise.

Exercise intensity is also an important determinant of the renal response. In healthy rats, a single bout of exhaustive treadmill exercise increased serum and renal miR-155, reduced renal SIRT1 expression, activated TXNIP/NLRP3 inflammasome signaling, and produced biochemical and histological evidence of AKI (Geng. et al., 2022). These findings indicate that exercise-associated miRNA remodeling may contribute to either physiological adaptation or renal stress depending on exercise dose and duration.

### 4.2 Metabolic and obesity-related kidney injury

Evidence linking renal miRNA remodeling to exercise-associated renal improvement has been reported in HFD-induced kidney injury. In HFD-fed mice, eight weeks of moderate treadmill exercise reduced proteinuria, renal injury markers, glomerular enlargement, mesangial expansion, inflammation, oxidative stress, apoptosis, and collagen deposition (Luan et al., 2025). Kidney miRNA sequencing identified 50 exercise-responsive miRNAs, indicating that the improvement in renal phenotype was accompanied by broad remodeling of the renal miRNA profile. Among the independently validated candidates, renal miR-150-5p and miR-182-5p were increased by HFD feeding and reduced following exercise (Luan et al., 2025). These changes occurred alongside improvements in oxidative balance, cell survival, and fibrotic remodeling, providing Level C evidence that regulation of these miRNAs accompanies the renoprotective response to exercise.

Evidence from non-exercise kidney injury models provides a potential mechanistic basis for these associations. Tubular-cell-derived EV miR-150-5p can promote fibroblast activation through SOCS1 suppression, whereas miR-182-5p increases ferroptotic susceptibility by targeting GPX4 (Ding et al., 2020; Zhou et al., 2021). These findings suggest that the exercise-associated reduction in miR-150-5p and miR-182-5p may be relevant to the attenuation of renal fibrosis and oxidative cell injury, although these specific pathways were not examined in the exercise study.

Other miRNAs detected in HFD kidneys may also participate in pathways modified by exercise. miR-802-5p, miR-30e-5p, and miR-542-3p have been implicated in NF-κB-dependent inflammation, Beclin 1-regulated autophagy, apoptosis, and AGO1-dependent fibrotic remodeling (Sun et al., 2019; Liu et al., 2021; Li et al., 2020). Their presence in HFD-associated renal miRNA profiles identifies additional candidate regulators through which exercise could influence inflammatory and reparative processes.

The limited overlap between the highest-ranked HFD-associated and exercise-responsive miRNAs suggests that exercise does not simply reverse disease-induced dysregulation (Luan et al., 2025). Instead, exercise-mediated renoprotection may involve a combination of normalization of pathogenic miRNAs, compensatory regulation, and the induction of exercise-specific adaptive responses.

### 4.3 Diabetic kidney disease

Exercise improves glucose handling, oxidative balance, renal histology, and kidney function in experimental DKD (Monno et al., 2021). Studies examining renal miRNAs indicate that the molecular responses accompanying these benefits vary according to the miRNA and experimental model.

In streptozotocin-induced diabetic rats, moderate exercise reduced renal miR-192 in healthy animals but did not clearly normalize its expression in diabetic kidneys (Oghbaei et al., 2015). miR-192 regulates TGF-β-dependent matrix production through cell- and context-specific interactions with ZEB-family repressors and downstream miR-200 signaling (Kato et al., 2007; Putta et al., 2012; Krupa et al., 2010). Its variable response to exercise suggests that miR-192 regulation depends on the metabolic and pathological state of the kidney.

Exercise also produced a modest, nonsignificant increase in renal miR-146a without coordinated changes in IRAK1, TRAF6, or downstream NF-κB signaling (Oghbaei et al., 2017). Although miR-146a can suppress renal inflammatory injury through IRAK1/TRAF6-dependent negative feedback, the available diabetic model does not indicate substantial activation of this pathway during exercise-induced renal improvement.

More integrated evidence has been reported for miR-126 and nuclear factor erythroid 2-related factor 2 (Nrf2)-related signaling. In diabetic rats subjected to myocardial IRI, exercise alone improved renal histology and catalase activity alongside increased renal miR-126 and Nrf2 expression. Combined treatment with Crataegus persica extract also improved selected redox indices and reduced urinary albumin and serum creatinine (Asgari et al., 2022). The concurrent regulation of the miRNA, antioxidant pathway, and renal phenotype provides Level B support for a potential contribution of miR-126/Nrf2 signaling to the protective effects of exercise.

### 4.4 Aging-related kidney injury

Aging-related kidney injury provides some of the most coherent evidence linking renal miRNA regulation with exercise-mediated improvements in fibrosis and metabolic homeostasis. In aged mice, aerobic exercise reduced renal p53 activation and miR-34a expression, increased Klotho, attenuated TGF-β/Smad and Wnt/β-catenin/matrix metalloproteinase 7 (MMP7) signaling, and reduced renal fibrosis (Zhao et al., 2024). The coordinated regulation of miR-34a, Klotho-centered signaling, and fibrotic outcomes supports a p53–miR-34a–Klotho pathway within the broader renoprotective response to exercise.

Swimming exercise in aged rats similarly altered renal miR-21 and miR-34a while restoring PPARα expression, reducing renal lipid accumulation, improving redox balance, and attenuating inflammatory and fibrotic signaling (Zhao et al., 2023). These findings place miR-21 and miR-34a within a wider exercise-induced response involving metabolic reorganization and suppression of maladaptive remodeling.

Additional Level B evidence has emerged from D-galactose-induced renal aging. Moderate-intensity endurance training and high-intensity interval training increased renal miR-296-5p, reduced SGLT2 protein abundance, and attenuated glomerulosclerosis. High-intensity interval training also improved serum creatinine and blood urea nitrogen (Mirtajaddini Goki et al., 2026). The corresponding changes in miR-296-5p, SGLT2, and renal outcomes support a potential role for the miR-296-5p–SGLT2 pathway in exercise-mediated protection against aging-related renal injury.

## 5 Potential contribution of extra-renal miRNAs to exercise-mediated renoprotection

The renoprotective effects of exercise may not be restricted to molecular events originating within the kidney. Exercise-responsive tissues, particularly skeletal muscle, release EVs and other circulating factors that may influence renal metabolism, inflammation, and fibrotic remodeling. This possibility is relevant because whole-kidney miRNA measurements cannot distinguish local transcriptional changes from the uptake of miRNAs produced by extra-renal tissues.

Studies of EV-associated miR-21a-3p provide mechanistic evidence for muscle-kidney communication. EVs from damaged or denervated muscle were enriched in miR-21a-3p and aggravated renal injury and fibrosis. In renal tubular cells, miR-21a-3p inhibition restored peroxisome proliferator-activated receptor-γ coactivator 1α (PGC-1α) expression and attenuated EV-induced injury (Jiang et al., 2025). In the exercise experiments, miR-21a-3p decreased in skeletal muscle, while plasma EVs collected after exercise attenuated TGF-β1-induced profibrotic responses in renal tubular cells. Similar effects were observed with plasma EVs from participants receiving resistance training combined with Korean mistletoe extract. These findings suggest a potential protective role for exercise-associated EVs, although the contribution of miR-21a-3p to this activity was not tested by miRNA-specific manipulation.

Complementary evidence for muscle-to-kidney miRNA transfer comes from non-exercise models. In streptozotocin-induced diabetic mice, intramuscular overexpression of miR-23a/27a increased the corresponding miRNAs in serum exosomes and kidney tissue and attenuated renal dysfunction and fibrosis (Zhang et al., 2018). Consistent with this, exosomal delivery of miR-29 to muscle resulted in renal accumulation and reduced kidney fibrosis through Yin Yang 1 (YY1)- and TGF-β-related mechanisms (Wang et al., 2019), while RVG-modified exosomes derived from skeletal-muscle satellite cells and carrying a miR-23a/27a/26a cluster accumulated in injured kidneys of db/db mice and alleviated tubular injury and tubulointerstitial fibrosis through suppression of *Lpp*, *Zbtb20*, and *Klhl42* (Ji et al., 2023). Together, these studies support the feasibility of muscle-to-kidney miRNA transfer, although they do not directly model physiological exercise.

## 6 Circulating and urinary miRNAs as biomarkers of exercise responses

Human studies have primarily examined miRNAs in plasma, serum, or urine. These biofluid miRNAs may help characterize individual responses to exercise, vascular adaptation, physical performance, and disease status in CKD, although they do not necessarily reflect miRNA regulation within the kidney.

In patients with nondialysis CKD, acute cardiopulmonary exercise increased plasma miR-150 in both patients and healthy participants, whereas miR-146a decreased selectively in participants with CKD (Van Craenenbroeck et al., 2015). miR-21, miR-126, and miR-210 showed no consistent acute responses. Following three months of aerobic training, resting plasma miRNA concentrations changed little, but the responses to a subsequent acute exercise bout were modified. Plasma miR-150 and miR-125b increased after acute exercise, while training altered the post-exercise response of miR-210. A greater exercise-induced reduction in miR-210 after training was associated with a greater improvement in peak oxygen uptake. Dynamic miRNA responses to an acute exercise challenge may therefore provide more information about training adaptation than resting concentrations alone.

In a separate cross-sectional study involving 63 patients with CKD and 18 healthy controls, circulating miR-146a and miR-150 were associated with estimated glomerular filtration rate, arterial stiffness, and peak oxygen uptake (Van Craenenbroeck et al., 2016). Their associations with peak oxygen uptake were attenuated after adjustment for pulse-wave velocity, linking these miRNAs more closely to vascular status and exercise capacity. The systemic origin of circulating miRNAs is important when comparing human biofluid studies with renal-tissue findings. Plasma miRNAs may arise from immune cells, platelets, vascular tissue, skeletal muscle, or other organs. Accordingly, the acute increase in circulating miR-150 does not conflict with the reduction in whole-kidney miR-150-5p after chronic exercise in HFD-fed mice. These responses arise from different biological compartments and may reflect distinct aspects of exercise adaptation.

Studies in patients receiving hemodialysis further illustrate the biomarker potential of circulating miRNAs. Baseline miR-206 predicted improvements in hip-flexion strength following intradialytic resistance training, whereas miR-23a was associated mainly with functional changes during physical inactivity (Spakova et al., 2020). In another study, 24 weeks of resistance training increased circulating miR-1 and reduced miR-31 alongside improvements in physical performance, inflammatory status, sestrin-2, and nitric oxide-related measures (de Luca Corrêa et al., 2022). These findings highlight circulating miRNAs as potential markers of systemic exercise adaptation in the dialysis setting. Their associations with physical performance, vascular measures, or inflammation, however, do not by themselves demonstrate improved kidney function or preservation of residual renal function.

Urinary EVs offer another noninvasive perspective on exercise-related molecular responses. In healthy young adults, acute exercise altered urinary EV abundance and miRNA cargo (Park and Moon, 2022). A separate cross-sectional study identified differences in urinary EV morphology, purine content, and metabolic miRNA profiles between endurance-trained triathletes and inactive controls (Pietrangelo et al., 2023). Although these studies did not involve kidney disease, they demonstrate the feasibility of detecting acute exercise-responsive and habitual training-associated EV-miRNA signatures in urine. Application of this approach in CKD may help determine whether urinary EV profiles track changes in albuminuria, glomerular filtration, renal injury, or responsiveness to exercise interventions.

## 7 Limitations and future directions

Current evidence linking exercise-associated miRNA remodeling to renal protection remains predominantly experimental and largely associative. Although several studies have reported coordinated changes in miRNA expression, related signaling pathways, and renal outcomes, the direct contribution of individual miRNAs to exercise-induced renoprotection has not been established. Moreover, most studies rely on whole-kidney measurements, which may obscure cell-specific responses and limit the identification of relevant cellular targets. Addressing these limitations will require well-defined exercise interventions combined with miRNA-specific gain- and loss-of-function approaches, renal structural and functional assessments, direct target validation, and rescue experiments. Spatially resolved or cell-specific analyses may further clarify the roles of candidate miRNAs across glomerular, tubular, endothelial, stromal, and immune compartments.

Interpretation and comparison of existing findings are further complicated by substantial heterogeneity in exercise protocols, disease models, and experimental populations. Exercise intensity, duration, training status, and underlying health conditions may influence the balance between renal adaptation and injury, as illustrated by the contrasting responses to regular training and acute exhaustive exercise. Differences in intervention timing and sample collection may also contribute to inconsistent miRNA findings across studies. Future investigations should therefore standardize the reporting of exercise protocols and sampling intervals, particularly the time elapsed between the final exercise session and tissue or biofluid collection. Consideration of sex and age, together with potential contributions from exercise-induced changes in body weight, glycemic control, and blood pressure, will also be important for interpreting renal outcomes and improving the generalizability of findings.

An additional limitation concerns the uncertain contribution of extra-renal miRNAs to exercise-mediated renoprotection. Although studies involving muscle-derived EVs support the biological feasibility of interorgan miRNA transfer, direct evidence that exercise-responsive EV-miRNAs reach the kidney and mediate protective effects remains limited. Future studies should identify the cellular origins of exercise-responsive EVs, characterize their miRNA cargo, trace their renal distribution and uptake, and determine whether selective manipulation of candidate EV-associated miRNAs alters renal outcomes. Parallel profiling of renal, circulating, EV-associated, and urinary miRNAs may help distinguish local expression from systemic release, renal uptake, and urinary excretion, thereby clarifying their respective contributions to renal adaptation and their potential value as biomarkers of exercise responses.

## 8 Conclusions

Available experimental evidence suggests that exercise-associated renal miRNA remodeling may be linked to improvements in renal outcomes through broader changes in cellular homeostasis, stress responses, and tissue remodeling. Among the candidate miRNAs, the miR-34a–Klotho, miR-296-5p–SGLT2, and miR-126/Nrf2 axes have relatively stronger same-study pathway support, whereas the potential contributions of miR-150-5p and miR-182-5p are supported primarily by associative findings and mechanistic evidence from non-exercise models. Beyond local renal responses, EV-associated miRNAs suggest a possible route for interorgan communication, while circulating and urinary miRNAs may serve as indicators of systemic exercise adaptation. Although direct causal mediation by individual miRNAs remains unproven, current findings highlight miRNA remodeling as a potential component of the molecular network linking exercise to renal protection.
